# Laparoscopic and Laparotomic Restaging in Patients With Apparent Stage I Epithelial Ovarian Cancer: A Comparison of Surgical and Oncological Outcomes

**DOI:** 10.3389/fonc.2022.913034

**Published:** 2022-06-20

**Authors:** Yongxue Wang, Jie Yin, Yan Li, Ying Shan, Yu Gu, Ying Jin

**Affiliations:** National Clinical Research Center for Obstetric & Gynecologic Diseases, Department of Obstetrics and Gynecology, Peking Union Medical College Hospital, Chinese Academy of Medical Sciences & Peking Union Medical College, Beijing, China

**Keywords:** laparoscopy, laparotomy, restaging, epithelial ovarian cancer, early stage

## Abstract

**Objective:**

To assess the surgical and oncological outcomes of laparoscopic restaging compared with laparotomy for apparent early-stage epithelial ovarian cancer.

**Methods:**

A retrospective chart review was undertaken of patients who underwent laparoscopic (laparoscopy group) or laparotomic (laparotomy group) restaging at the Peking Union Medical College Hospital, China, between January 2012 and December 2017. All patients had apparent stage I epithelial ovarian cancer that was incompletely staged at the initial surgery.

**Results:**

A total of 157 patients were included, with 50 in the laparoscopy group and 107 in the laparotomy group. Baseline characteristics were similar between the groups. No cases were converted from laparoscopy to laparotomy. The laparoscopy group had a significantly shorter operating time (p<0.001), less estimated blood loss (p<0.001), and a shorter postoperative hospitalization duration (p<0.001) than the laparotomy group. Transfusions were required in only eight laparotomy patients. No significant differences in postoperative complications were observed between the two groups (p=0.55). Eighteen (11.5%) patients were upstaged to stage II or stage III after surgery. A total of 123 (78.3%) patients received postoperative platinum-based chemotherapy. During the follow-up period, 15 (9.6%) patients experienced disease recurrence, and 3 patients died of disease progression. Five-year disease-free survival (p = 0.242, log-rank test) and overall survival (p = 0.236, log-rank test) were not affected by the surgical approach.

**Conclusions:**

Laparoscopic restaging showed more favorable operative outcomes than laparotomy. Surgical restaging *via* laparoscopy versus laparotomy was not associated with worse survival in women with apparent stage I epithelial ovarian cancer.

## Introduction

Epithelial ovarian cancer (EOC) is a leading cause of cancer-related mortality among women worldwide: it is estimated that in 2018, almost 185,000 deaths from OC occurred globally (1). Most epithelial ovarian cancers are detected at an advanced stage because of the lack of screening methods and specific symptoms, with early-stage epithelial ovarian cancer accounting for only 20-25% of cases (2, 3). The diagnosis of early-stage epithelial ovarian cancer often occurs due to accidental findings, and unfortunately, the current preoperative assessment of adnexal masses using imaging and serum tumor markers such as CA125 does not allow for the detection of early-stage ovarian cancer with sufficient accuracy (4).

Complete staging surgery for early-stage epithelial ovarian cancer includes hysterectomy, bilateral salpingo-oophorectomy (BSO), omentectomy, peritoneal biopsy, pelvic and para-aortic lymph node dissection, and peritoneal washings to identify occult, advanced-stage disease. However, early-stage epithelial ovarian cancer is often diagnosed during the removal of begin-appearing ovarian tumors, so a surgeon with the skills to perform a surgical-staging procedure might not be present. For patients who do not undergo complete staging surgery at the time of the initial surgery, a restaging procedure is essential to obtain prognostic information (5). This information is particularly important for guiding decisions regarding whether to withhold or recommend adjuvant treatment and maintenance therapy. Up to 30% of women with apparent early-stage disease have microscopic metastasis (5–7). Bae et al. (7) conducted a study including 14 patients to evaluate the feasibility of laparoscopic restaging surgery and found that 28.6% of patients were upstaged. A study by Hengeveld et al. (8) found that the proportions of lymph node and greater omentum metastases in early-stage ovarian cancer were 4.7% and 3.7%, respectively.

Minimally invasive surgery (MIS) has been widely used to treat gynecologic malignancies. It is the standard surgical approach for endometrial cancer, and some clinical studies have been conducted to explore whether interval debulking surgery should be performed after neoadjuvant chemotherapy for advanced ovarian cancer (9–11). However, minimally invasive surgery for early-stage ovarian cancer remains controversial. Many studies have found that MIS increases the risk of ovarian tumor rupture, thereby affecting patient staging and prognosis (12, 13). However, some studies have shown no difference in surgical outcomes, recurrence rates, or survival between those who underwent minimally invasive surgery and those who underwent open surgical staging (9, 14). A systematic Cochrane review suggested that there is not enough good evidence to quantify the risks and benefits of laparoscopy for the management of early-stage EOC (15). The National Comprehensive Cancer Network (NCCN) guidelines for ovarian cancer recommend that the use of MIS for primary surgical treatment of early-stage ovarian cancer should be limited to selected patients and performed by experienced surgeons (16). For apparent early-stage EOC patients with incomplete staging, the pelvic mass is removed at the initial surgery so that there is no risk of capsule rupture when restaging surgery was performed. Whether minimally invasive surgery increases the complications of restaging surgery and whether it affects patient prognosis are unclear. The purpose of this study was therefore to investigate the surgical and oncological outcomes of laparoscopic restaging compared with laparotomy for apparent early-stage epithelial ovarian cancer patients with incomplete staging at initial surgery.

## Materials and Methods

### Patients

We performed a retrospective analysis involving patients undergoing restaging surgery for apparent early-stage ovarian cancer who were incompletely staged at the time of initial surgery between 2012 and 2017 at Peking Union Medical College Hospital (PUMCH), Beijing, China. The patients included in this study were those who underwent initial surgery at our hospital or were referred to our hospital after their initial surgery. The study design was approved by the Institutional Review Board (IRB) of PUMCH. Each patient signed a consent form for data collection for research purposes.

The eligibility criteria were as follows: (1) age 18 years or older; (2) an Eastern Cooperative Oncology Group performance status ≤2; (3) invasive epithelial ovarian cancer confirmed by two pathologists; (4) macroscopic spread not observed during the initial surgery; (5) a negative pre-operative CT scan for positive nodes (defined as lymph nodes <1 cm in their larger axis); (6) incompletely staged at the initial surgery and (7) interval between the initial surgery and restaging surgery of less than 90 days. Patients were excluded in case of evidence of carcinomatosis; borderline tumors; received chemotherapy prior to restaging surgery; a history of a malignant tumor in the abdominal cavity; previous abdominal therapy.

### Surgical Procedures

Laparoscopic staging procedures had to be performed by surgeons with extensive training in gynecologic oncology and minimally invasive surgery. Patients were placed in the Trendelenburg position. A pneumoperitoneum was created by inserting a Veress needle through the umbilicus and introducing CO2 gas to 14 mmHg. Five trocars were used: 10-mm laparoscopic ports were placed in the umbilicus, left iliac fossa and left upper quadrant, and 5-mm ports were placed in the right iliac fossa and suprapubic area.

The staging surgery was performed according to the procedures of Bae et al (7). The laterocaval, precaval, and interaorticocaval and lateroaortic nodal groups were resected from the left renal vein cranially to include bilateral pelvic node dissections caudally In some cases, the upper level of para-arotic lymph node dissection was inferior mesenteric artery. In most of the patients, the omentectomy was infragastric as follows. The avascular portion of the omental attachment to the transverse colon was removed, and the gastrocolic ligament transected using LigaSure (Covidien, Boulder, CO, USA). After the omentum was divided from the transverse colon and stomach, it was placed into an endobag for removal. An appendectomy was performed optionally.

A total hysterectomy with salpingo- oophorectomy was then performed totally laparoscopically. After removing the appendix, uterus, adnexa, and omentum within an endobag, the vaginal stump was closed using laparoscopic intracorporeal interrupted sutures. Any suspicious growth was biopsied. In the case of normal visual exploration, random peritoneal biopsies were performed in the Douglas pouch, pelvic and abdominal parietal peritoneum, paracolic gutters, hemidiaphragms, and mesentery. Drainage tubes were inserted *via* a 5-mm trocar on both sides.

For laparotomy surgery, the preoperative preparation, surgical procedures, and postoperative management were essentially the same as for the laparoscopic approach, except that a midline vertical incision from the pubic symphysis to the xiphoid process was created.

Fertility sparing surgery (FSS) was performed for some young (age < 40 years) patients who wished to preserve their childbearing potential. Usually, this surgery consists of preservation of the uterus and the contralateral adnexa. Lymph node dissection can be omitted for early-stage mucinous carcinoma.

### Data Collection

Data on the characteristics of the patients, surgical procedures, histological findings, and follow-up data were obtained from the medical records. Patients were divided into the laparoscopy group and laparotomy group according to the surgical approach used for restaging. The baseline characteristics of the patients included age, body mass index (BMI), time interval between initial surgery and restaging surgery, initial surgical approach, procedures at initial surgery, whether rupture of the capsule occurred during the initial surgery, and pathologic type of tumor.

Surgical outcomes included the operative time, estimated blood loss, surgical procedures used, postoperative hospitalization duration, final FIGO stage, presence of upstaging, postoperative complications, postoperative adjuvant treatment options, follow-up time and disease status. The postoperative hospitalization duration was calculated starting from the first day after surgery. Postoperative complications were defined as events occurring within 30 days after the surgery. In-hospital postoperative complications were recorded from the medical records, whereas complications that occurred after discharge were recorded during follow-up visits. All patients treated prior to 2014 were restaged to the 2014 Federation of Gynecology and Obstetrics (FIGO) staging system for ovarian, fallopian tube and peritoneal cancer based on the findings during surgery and *via* pathology (3).

Patients were followed up regularly after surgery in accordance with the NCCN guidelines (16). Follow-up was scheduled every 3 months for 2 years, then every 6 months for 3 years, and annually thereafter. Disease-free survival (DFS) was calculated from the date of the initial surgery to the date of recurrence or the date of the last follow-up. Overall survival (OS) was calculated from the date of the initial surgery to the date of death or the date of the last follow-up.

### Statistical Analysis

The t test and Mann–Whitney test were used to compare continuous variables as appropriate. Chi-square and Fisher exact tests were used for categorical variables. Survival outcomes were estimated using the Kaplan–Meier model. The log-rank test was used to compare the risk of developing recurrence and the risk of death between the 2 groups over time. A p<0.05 was considered statistically significant. Data were analyzed using SPSS software for Windows (version 20.0; SPSS Inc., Chicago, IL).

## Results

### Clinical Features of Patients

Overall, 182 patients with apparent stage I epithelial ovarian cancer underwent restaging surgery at PUMCH between 2012 and 2017. Twenty-five patients did not meet the criteria for inclusion, 10 of whom received chemotherapy before restaging surgery, 8 lacked complete data, and 7 had borderline tumors. A total of 157 patients were included, with 50 patients in the laparoscopy group and 107 patients in the laparotomy group.

The characteristics of the patients are shown in **Table 1**. The differences in patient characteristics between the laparoscopy group and laparotomy group were nonsignificant for all criteria except parity. More women were nulliparous in the laparoscopy group than in the laparotomy group (48% vs. 29.9%, p=0.023).

**Table 1:** Patient characteristics.

Variable	Laparoscopic group n (%) N=50	Laparotomy group n (%) N=50	P-value
Age, mean (SD), y	35.0 (11.2)	38.5 (10.9)	0.072
BMI, median (range)	22.3 (14.9,29.4)	21.8 (17.5,29.4)	0.881
Maximum diameter of tumor^&^, cm	8 (6,12)	8 (6,11)	0.883
Interval between initial and restaging surgery^&^, day	40 (18-79)	38 (14-89)	0.577
Nulliparous	24 (48.0%)	32 (29.9%)	0.023
Initial surgical approach, n (%)			0.207
Laparoscopy	26 (52.0%)	67 (62.6%)	
Laparotomy	24 (48.0%)	40 (37.4%)	
Histology			0.658
Serous	10 (20.0%)	16 (14.9%)	
Clear cell	12 (24.0%)	35 (32.7%)	
Endometrioid	10 (20.0%)	24 (22.4%)	
Mucinous	17 (34.0%)	31 (29.0%)	
Mixed	1 (2.0%)	1 (0.93%)	
Grade			0.181
G1	29 (58.0%)	51 (47.7%)	
G2	1 (2.0%)	10 (9.3%)	
G3	20 (40.0%)	46 (43.0%)	
Procedures used in the initial surgery			0.052
USO	23 (46.0%)	61 (57.0%)	
Cystectomy	20 (40.0%)	29 (27.1%)	
TH+BSO	5 (10.0%)	17 (15.9%)	
TH+USO	2 (4.0%)	0 (0.00)	

USO, Unilateral salpingo-oophorectomy; BSO, Bilateral salpingo-oophorectomy; TH, Total hysterectomy; ^&^Median (range).

### Surgical Outcomes

**Table 2** summarizes the surgical outcomes. The length of postoperative hospitalization duration was significantly shorter in the laparoscopy group (p < 0.001). The operative time was significantly longer in the laparotomy group than in the laparoscopy group (p <0.001); the amount of estimated blood loss was higher in the laparotomy group than in the laparoscopy group (p <0.001). Eight patients required intraoperative or postoperative transfusion in the laparotomy group, while none required transfusion in the laparoscopy group (p =0.047). In addition, no patients underwent conversion to laparotomy in the laparoscopy group. Postoperative complications occurred in 3 and 10 patients in the laparoscopy and laparotomy groups, respectively, but this difference was not significant (6.0% vs. 9.3%, p = 0.55).

**Table 2:** The surgical outcomes of patients.

Variable	Laparoscopic group n (%)	Laparotomy group n (%)	P-Value
Total number of patients	50	107	
Hospitalization duration^&^, day	6 (3,33)	8 (2,23)	<.0001
Operative time^&^, min	150 (60,300)	180 (150,200)	0.0015
Estimated blood loss^&^, ml	100 (20,400)	200 (100,1500)	<.0001
Blood transfusion required	0 (0.00)	8 (7.48%)	0.0472
Postoperative complications	3 (6.0%)	10 (9.3%)	0.553
Type of postoperative complications			
Deep venous thromboembolism	0	2	
Infections	2	3	
Bowel obstruction	0	4	
Would dehiscence	0	1	
Lymphorrhea	1	0	
Fertility-sparing surgery	28 (56.0%)	24 (22.4%)	<0.0001
Pelvic lymphadenectomy			0.138
No	1 (2.0%)	0 (0.00)	
Yes	48 (98.0%)	107 (100.0%)	
Para-aortic lymphadenectomy			0.001
No	27 (54.0%)	29 (27.1%)	
Yes	23 (46.0%)	78 (72.9%)	
Plevic node count^&^	26 (8-45)	26 (8-48)	0.089
Para-aortic node^&^	5 (1-26)	7 (1-33)	0.216
Definitive FIGO stage			0.805
IA	7 (14.0%)	12 (11.2%)	
IC	38 (76.0%)	82 (76.6%)	
II	2 (4.0%)	8 (7.5%)	
III	3 (6.0%)	5 (4.7%)	

^&^Median (range).

There were more patients who underwent fertility-sparing surgery in the laparoscopy group than in the laparotomy group (56% vs. 24%, p<0.0001). In the whole series, only 1 patient did not undergo pelvic lymphadenectomy. A total of 101 (64.3%) patients underwent para-aortic lymphadenectomy. Compared to the laparoscopy group, more patients underwent para-aortic lymphadenectomy in the open surgery group (72.9% vs. 46%, p=0.001). The median number of pelvic and para-aortic lymph nodes retrieved was similar between the laparoscopy and laparotomy groups (26 vs. 26, respectively; 5 vs. 7, respectively).

Overall, 18 of 157 patients (11.5%) were upstaged to stage II or stage III, with 5 patients in the laparoscopy group and 13 patients in the laparotomy group (10% vs. 12.1%, p=0.69). Ten patients were upstaged to stage II due to pathological findings on the surface of the pelvic peritoneum. Upstaging to stage III was due to malignant spread to the pelvic lymph nodes.

### Survival Outcomes

**Figure 1:**
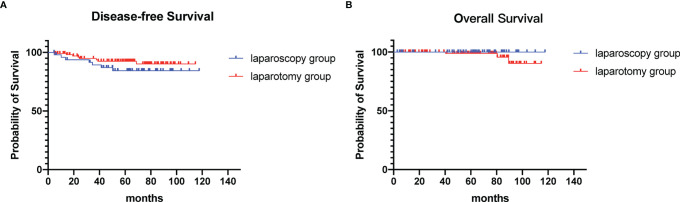
Survival outcomes of patients. **(A)** Disease-free survival **(B)** Overall survival.

The treatment method and survival outcomes are shown in **Table 3**. Overall, 123 (78.3%) patients received postoperative platinum-based chemotherapy, namely, 37 in the laparoscopic group and 86 in the open group, with no significant difference observed between the two groups (74% vs. 80.4%, p=0.34). The median follow-up times were 63.6 ± 27.6 months and 61.8 ± 28.2 months in the laparoscopy group and laparotomy group, respectively. During the follow-up period, 15 (9.6%) patients experienced disease recurrence, with 7 in the laparoscopy group and 8 in the laparotomy group. Three patients died of disease progression, all of whom were in the laparotomy group. The five-year disease-free survival rate and overall survival rate were 84.4% and 100% in the laparoscopy group vs. 92.7% and 98.8% in the laparotomy group, respectively. Disease-free survival (p= 0.242, log-rank test) and overall survival (p= 0.236, log-rank test) were not affected by the surgical approach (**Figure 1**).

**Table 3:** Postoperative treatment methods and survival outcomes.

Variable	Laparoscopy N (%)	Laparotomy N (%)	P-value
Total number of patients	50	107	
Treatment			0.34
Surgery only	13 (26.0%)	21 (19.6%)	
Surgery+adjuvant chemotherapy	37 (74.0%)	86 (80.4%)	
Paclitaxel/carboplatin	33 (66%)	85 (79.4%)	
Cyclophosphamide/carboplatin	1 (2.0%)	1 (0.93%)	
Paclitaxel/cisplatin	3 (6.0%)	0	
Follow-up period, mean ± SD (mo)	63.6 ± 27.6	61.8 ± 28.2	0.78
Tumor recurrence	7 (14.0%)	8 (7.5%)	
Death from disease	0	3 (2.8%)	

## Discussion

Our results have provided evidence that laparoscopic restaging might be adequate and feasible for the treatment of apparent early-stage ovarian cancer who were incompletely staged at the time of initial surgery. The laparoscopic restaging showed more favorable operative outcomes than laparotomy. The operative time, postoperative hospital stays, and intraoperative blood loss were significantly lower in the laparoscopy group. In addition, there was no difference in the surgical complication rate between the laparoscopy group and the laparotomy group. However, five-year disease-free survival and overall survival were not affected by the surgical approach.

Early-stage epithelial ovarian cancer has a very good prognosis, with 90% 5-year overall survival (4). However, the preoperative diagnosis of early-stage ovarian cancer is difficult, so it is often detected incidentally by intraoperative or postoperative pathology. Restaging surgery is essential for these patients, as it serves as a guide for subsequent adjuvant therapy (17), and the upstaging rate of apparent stage I can even be as high as 32% (0-41.7%) (8, 18–21). In addition, a study by Bizzarri et al. found that staging lymphadenectomy represented an independent factor which improves 5-year disease-free survival in apparent early-stage epithelial ovarian cancer (22). Observation is an option after restaging surgery if the results confirm stage IA/IB disease. Maintenance therapy can be applied after the completion of adjuvant chemotherapy in patients with stage II-IV disease after full staging has been completed. Leblanc et al. (5) reported that 19% of patients were upstaged after restaging surgery. Bogani et al. (23) reported that approximately 15% of early-stage ovarian cancers were diagnosed with positive nodes. High-grade serous tumors and bilateral tumors were the main characteristics suggesting lymph node positivity. Hengevel et al. (8) studied the value of surgical staging in patients with apparent early-stage epithelial ovarian cancer. They found that one-third of patients were upstaged, and one-third of upstaged patients had an altered treatment plan. Upstaging occurred in 15.9% of patients due to peritoneum, omentum and retroperitoneal lymph node involvement. In our study, the rate of upstaging (11.5%) was similar to that in other studies.

Many case series studies have confirmed the safety of laparoscopic staging surgery for early-stage ovarian cancer (5, 6, 10). In the present study, there was no significant difference in the incidence of 30-day postoperative complications between the laparoscopy group and the laparotomy group. This is consistent with the results of previous studies. In a study by Melamed et al. (24) conducted with data from the National Cancer Database, the frequency of surgical complications within 90 days of surgery and unplanned readmission within 30 days did not differ between the planned laparoscopic staging group and the planned laparotomy staging group. In a case–control study by Dito, the complication rate was similar between the minimally invasive surgical staging and open surgery groups, and there was no conversion to open surgery in the minimally invasive group (14).

Furthermore, the NCCN guidelines recommend that minimally invasive surgery be performed by a gynecologic oncologist experienced in minimally invasive surgery (16). The biggest concern during laparoscopic staging surgery is tumor rupture; however, in secondary staging procedures, there is no risk of intraoperative mass rupture since the tumor was removed during the first procedure. ESMO-ESGO recommends that a minimally invasive approach be considered for restaging surgery (25).

Patients in the laparoscopy group had a significantly shorter postoperative hospitalization duration than those in the open group. In the present study, the median postoperative hospitalization duration was significantly shorter in the laparoscopy group (p<.0001). In the study by Park et al. (6), the laparoscopic group had a shorter hospitalization duration than the laparotomy group. Melamed et al. (24) conducted a case–control study for stage I epithelial ovarian cancer and found that patients who underwent laparoscopic surgery had shorter postoperative stays and higher lymph node counts. There was no significant difference in the overall survival between women who underwent laparoscopic staging and those who underwent laparotomy staging.

The retroperitoneal lymph node count can, to some extent, reflect the quality of surgery. Many studies have shown that the number of retroperitoneal lymph nodes evaluated in early-stage ovarian cancer patients undergoing minimally invasive surgery is not less than that evaluated in patients undergoing open surgery (4, 24). There was no difference in the number of lymph nodes in the two groups in the present study. Melamed et al. (24) found that patients who underwent laparoscopy had more lymph nodes excised than those who underwent laparotomy, suggesting that staging quality was not inferior.

Laparoscopic restaging surgery did not affect the oncological prognosis. In the present study, there was no difference in median DFS and OS between the laparoscopic and open surgery groups. This is also consistent with the results of previous studies. A published meta-analysis that included 3065 cases of early-stage ovarian cancer showed that survival outcomes were not influenced by the route of surgery (21). In a study by Melamd and colleagues using the National Cancer Database, the author reported that surgical staging *via* planned laparoscopy versus laparotomy was not associated with worse survival in women with apparent stage I epithelial ovarian cancer (HR=0.82, 95% CI 0.57-1.16) (9). Moreover, with the advancement of minimally invasive surgical techniques, minimally invasive surgery has also started to be used for interval debulking surgery in advanced ovarian cancer after neoadjuvant chemotherapy (9).

Sentinel lymph node (SLN) dissection is a new issue in staging surgery for apparent early-stage epithelial ovarian cancer. Lymphatic mapping for the assessment of SLNs is a widely accepted part of the surgical treatment of endometrial and cervical cancer (26). Two clinical trials are currently ongoing to clarify the use of sentinel lymph node technique in early ovarian cancer: SELLY (Sentinel Lymph Node in Early Ovarian Cancer, NCT03563781) (27) and SENTOV (Sentinel Lymph Node Technique in Early Ovarian Cancer, NCT03452982) (28). The preliminary results of SELLY trial revealed that the detection rate of SLN was 67.7%. In patients with lymphatic dissemination, the sensitivity and negative predictive value were 100% (29). Both trials confirmed the feasibility of SLN. The one-step nucleic acid amplification (OSNA) method is a promising emerging technique as one of the sentinel node biopsy techniques (30). It could identify lymph node metastasis during surgery, thus avoiding a second surgery. The feasibility and accuracy of the OSNA method in SLN mapping of gynecologic cancer were validated (31, 32). SLN in early-stage ovarian cancer has the potential to provide reliable and useful information on nodal status and may allow the avoidance of systematic lymphadenectomy in the majority of patients.

The present study presents different strengths:strict inclusion and exclusion criteria to obtain the most uniform population possible, with sufficient sample size and power for the study; all patients were epithelial ovarian cancer; all patients were undergone incompletely staging surgery previously; all patients were treated in the same center. However, the present study also has certain weaknesses. This study was retrospective in nature, and due to the characteristics of retrospective studies, selection bias could occur. Some patients who were referred to our center after their initial surgery had missing data. Moreover, the long inclusion period is a limitation of the study. These factors could affect the validity of this study to some extent.

## Conclusion

In conclusion, this study showed that laparoscopic restaging showed more favorable operative outcomes than laparotomy when performed by surgeons with considerable experience in laparoscopic surgery for gynecological malignancy. There was no difference in the oncologic outcomes of patients undergoing laparoscopic restaging compared with open restaging. Large prospective studies comparing the 2 approaches are warranted to confirm these findings.

## Data Availability Statement

The original contributions presented in the study are included in the article/supplementary material. Further inquiries can be directed to the corresponding author.

## Ethics Statement

The studies involving human participants were reviewed and approved by the Institutional Review Board (IRB) of PUMCH. The patients/participants provided their written informed consent to participate in this study.

## Author Contributions

YW: study concepts, literature research, clinical studies, data collection and analysis, manuscript writing and review. JY: study design, literature research, data collection, manuscript writing and review. YS: study design, data collection and analysis, manuscript writing and review. YG: data acquisition, manuscript preparation and data analysis. YJ: study design, literature research, data collection, manuscript writing and review. YL: study concepts, literature research, data collection and analysis, manuscript writing and review. All authors have read and approved the submission of the manuscript.

## Conflict of Interest

The authors declare that the research was conducted in the absence of any commercial or financial relationships that could be construed as a potential conflict of interest.

## Publisher’s Note

All claims expressed in this article are solely those of the authors and do not necessarily represent those of their affiliated organizations, or those of the publisher, the editors and the reviewers. Any product that may be evaluated in this article, or claim that may be made by its manufacturer, is not guaranteed or endorsed by the publisher.
